# Editorial: The Role of Sphingolipid Metabolism in the Development of Type 2 Diabetes and Obesity

**DOI:** 10.3389/fendo.2021.835751

**Published:** 2022-01-07

**Authors:** Agnieszka Blachnio-Zabielska, Eric Hajduch, Hervé Le Stunff

**Affiliations:** ^1^ Epidemiology and Metabolic Disorders Department, Medical University of Bialystok, Bialystok, Poland; ^2^ Centre de Recherche des Cordeliers, INSERM, Sorbonne Université, Paris, France; ^3^ Université Paris-Saclay, CNRS UMR 9197, Institut des Neurosciences Paris-Saclay, Saclay, France

**Keywords:** sphingosine-1-phosphate, ceramides, lipotoxicity, ApoM, biomarkers, type 2 diabetes

## Introduction

Sphingolipids are a group of biologically active lipids that are involved in the regulation of many cellular processes, including proliferation, apoptosis, and cellular differentiation (1). These sphingolipids include, among others, ceramides and sphingosine-1-phosphate (S1P), two lipids that usually play an opposite role in the above processes. For over twenty years, researchers’ attention has been focused on the role of these lipids in the onset of obesity-related insulin resistance (IR), which is the basis of type 2 diabetes (T2D) (2, 3). The aim of the Research Topic was to gather the latest knowledge on the role of sphingolipids in the development of obesity-related T2D and its metabolic complications.

In a mini-review by Juchnicka et al., the authors presented the current state of knowledge of both ceramide and S1P in the induction of IR in skeletal muscles and adipose tissue. The authors highlighted the role of sphingolipids in adipose tissue, which does not play a dominant role in insulin-dependent glucose uptake, but seems to play a critical role in induction of systemic glucose disturbances. Adipose tissue is not only a site of triacylglycerol accumulation, but also an important endocrine tissue. As such, adipocytokines secreted by adipose tissue, such as adiponectin, TNF-α and resistin, can influence insulin sensitivity in muscles and liver. Authors emphasized that ceramide content in different adipose tissue depots changes with obesity. They cited a work in which ceramide synthase 6 (CerS6) knock-out mice, despite high fat feeding, displayed a reduced body weight, adipose tissue content, reduced adipocyte size and improved glucose metabolism, suggesting that C16:0-ceramide, a product of CerS6, is a significant factor in the development of obesity. The article also presents conflicting data on the role of S1P in the pathogenesis of IR and obesity-related inflammation.

A topic related to S1P was the subject of another article on the role of sphingosine kinase (SphK) in the development of T2D. SphK is an enzyme that catalyzes the phosphorylation reaction of sphingosine to produce S1P. This enzyme affects not only S1P concentration, but also ceramide content. Qi et al. pointed out that SphK, due to the fact that it directly synthetizes S1P, a lipid that usually performs opposite functions to ceramide, acts as a switch of sphingolipid rheostat. Interestingly, the two isoforms of this enzyme, SphK1 and SphK2, display a different expression tissue profile, different subcellular location and to some extent, can play opposite functions, especially in terms of their influence on inflammation, diabetes and apoptosis. SphK exerts biological effects, mainly through its enzymatic product - S1P, and the diversity of S1P activities results, inter alia, from the fact that S1P acts on cell metabolism through five specific G protein-coupled membrane receptors, as well as by binding directly to certain intracellular protein domains, causing a change in their activity.

Another work on this Research Topic by Christoffersen, concerns the apolipoprotein M/S1P (apoM/S1P) complex and its role in processes related to the induction of IR, as well as the potential use of this complex as a diabetes biomarker. ApoM is an important carrier of circulating S1P, mainly associated to HDL. This complex is believed to play an important role in cholesterol metabolism, in regulating inflammatory processes, and in maintaining a healthy endothelial barrier, thus reducing cholesterol accumulation in vessel walls. Unfortunately, data on the role of ApoM in the development of diabetes are inconclusive, since the author cited works showing that, on the one hand, ApoM/S1P has a beneficial effect on insulin sensitivity, but on the other hand that ApoM deficiency is associated with protection against diet-induced obesity. The author presented the possibility of a potential use of ApoM as a biomarker that distinguishes patients with diabetes MOODY3 from type 1 diabetes (T1D), but not T2D.

Another paper by Mah et al. concerns the possibility of using circulating ceramides as biomarkers of cardiovascular diseases, atherosclerosis and diabetes. It has been proven many times that the concentration of circulating ceramide increases in obese patients, in patients with T2D and in patients with cardiovascular diseases compared to control individuals. A large-scale cohort study evaluating diabetes outcomes showed that serum C16:0-ceramide and C18:0-ceramide positively correlated with IR, therefore these ceramide species could be used as biomarkers of IR. The authors presented a thorough review of research on the possibility of using circulating ceramides as biomarker of metabolic disorders.

One of the most common comorbidities of diabetes is diabetic kidney disease (DKD). The review by Nicholson et al., described the available literature on the contribution of biologically active lipids in the development of DKD. The authors noted that the profile of circulating sphingolipids differed in people with DKD compared to diabetic controls. They presented a wide range of data indicating that changes in sphingolipid levels were associated with the development of diabetic nephropathy. In addition, they emphasized that it has not yet been established whether these changes in serum sphingolipid levels were due to altered sphingolipid metabolism in the kidney or a consequence of altered metabolism in other tissues.

Beyond a well-recognized direct effect of bioactive lipids on the insulin signaling pathway, sphingolipids were also shown as potent modulators of mitochondrial function. The paper by Roszczyc-Owsiejczuk and Zabielski discuss various aspects of mitochondrial insufficiency observed at the early and advanced IR stages in relation to sphingolipid content. Authors cited recent findings regarding the impact of sphingolipid accumulation on the impairment of the respiratory chain efficiency, mitochondrial ATP production and reactive oxygen species excessive generation. Authors argue that various mitochondrial abnormalities, such as decreased mitochondrial oxidative capacity and disturbed intracellular energy balance, are associated with specific classes of sphingolipids in a key aspect of mitochondrial metabolism.

In summary, articles presented in the Research Topic provide a valuable source of information concerning the direct role of ceramides and S1P in influencing many cellular functions and diseases such as insulin sensitivity, mitochondrial function, T2D, and diabetic kidney disease. In addition, it points out a possible and important role of circulating sphingolipids as potential biomarkers for the early detection of cardiovascular disease and T2D.

## Author Contributions

All authors listed have made a substantial, direct and intellectual contribution to the work, and approved it for publication.

## Funding

This study was supported by INSERM (EH), the Fondation de France and the Société Française du Diabète (EH and HLS), Centre National de la Recherche Scientifique (HLS) and the Foundation for Polish Science, grant TEAM/2016-1/2 (ABZ).

## Conflict of Interest

The authors declare that the research was conducted in the absence of any commercial or financial relationships that could be construed as a potential conflict of interest.

## Publisher’s Note

All claims expressed in this article are solely those of the authors and do not necessarily represent those of their affiliated organizations, or those of the publisher, the editors and the reviewers. Any product that may be evaluated in this article, or claim that may be made by its manufacturer, is not guaranteed or endorsed by the publisher.
